# Prevalence and associated factors of subjective cognitive decline (SCD Plus): a cross-sectional analysis of three population-based European cohorts

**DOI:** 10.1186/s13195-026-02087-4

**Published:** 2026-05-22

**Authors:** Andrea E. Zülke, Melanie Luppa, Christian Sander, Ronny Baber, Ronald Biemann, Kerstin Wirkner, Samira Zeynalova, Silke Zachariae, Milica Matijevic, Christoph Engel, Nigar Reyes, Andreas Hinz, Heide Glaesmer, Matthias L. Schroeter, A. Veronica Witte, Arno Villringer, Markus Löffler, Carol Brayne, Steffi G. Riedel-Heller

**Affiliations:** 1 Institute of Social Medicine, Occupational Health and Public Health, University of Leipzig, Leipzig, Germany; 2 Department of Psychiatry and Psychotherapy, University Hospital Leipzig, Leipzig, Germany; 3 Institute of Laboratory Medicine, Clinical Chemistry and Molecular Diagnostics, University Hospital Leipzig, Leipzig, Germany; 4 Leipzig Research Centre for Civilization Diseases, Leipzig University, Leipzig, Germany; 5 Institute for Medical Informatics, Statistics and Epidemiology, Leipzig University, Leipzig, Germany; 6 Department of Medical Psychology and Medical Sociology, Leipzig University, Leipzig, Germany; 7 Department of Neurology, Max Planck Institute for Human Cognitive and Brain Sciences, Leipzig, Germany; 8 Clinic for Cognitive Neurology, University Hospital Leipzig, Leipzig, Germany; 9 Department of Public Health and Primary Care, University of Cambridge, Cambridge, UK

**Keywords:** Subjective cognitive decline, Ageing, Alzheimer’s disease, Population-based study, Prevalence, Comorbidity

## Abstract

**Background:**

Subjective cognitive decline (SCD) is considered an early preclinical marker for Alzheimer’s disease (AD). However, prevalence estimates vary widely due to inconsistent definitions. SCD Plus criteria were proposed by experts to improve specificity for preclinical AD. Population-based evidence on the prevalence and correlates of SCD Plus remains limited.

**Methods:**

Data from three population-based European cohorts (LIFE-Adult-Study, English Longitudinal Study of Ageing, and Cognitive Function and Ageing Study) comprising adults aged ≥ 60 years without dementia or marked cognitive impairment were harmonized to derive a common definition of SCD Plus based on available core criteria. Generalized linear models examined correlates of SCD Plus in pooled and study-specific analyses.

**Results:**

Among 18,795 participants (mean age (SD): 72.1 (6.8) years; 55.5% women), prevalence of SCD Plus, based on the harmonized operationalization, was 37.9% (33.3–53.7% across cohorts). In pooled analyses, SCD Plus was associated with higher education, depression, anxiety, hypertension, diabetes, heart disease, Parkinson’s disease, and history of stroke, and inversely associated with smoking and better cognitive performance. Study-specific analyses additionally indicated associations with sleep and hearing-related problems, lower physical activity, thyroid disease, and personality traits (higher neuroticism and lower openness, agreeableness, conscientiousness).

**Discussion:**

SCD Plus was highly prevalent in three large European cohorts, assessed using a harmonized operationalization approach. Associations with several established, modifiable dementia risk factors underscore the relevance of SCD Plus for clinical risk assessment. Longitudinal studies are needed to determine whether the identified correlates influence subsequent cognitive decline in individuals with SCD Plus.

**Supplementary Information:**

The online version contains supplementary material available at 10.1186/s13195-026-02087-4.

## Background

The older population is growing worldwide, which brings about increasing prevalence of Alzheimer’s disease (AD) and other forms of dementia. Pathophysiology of AD can occur beyond a decade before objective cognitive decline becomes apparent. While two new amyloid antibody therapies have been found successful in decreasing amyloid beta and slowing disease-related cognitive decline in patients with mild cognitive impairment (MCI) and early AD [1, 2], a cure for relatively early onset dementia of pure Alzheimer’s form (minority of dementia in older populations) is currently not on the horizon, and research has focused on early and preclinical stages of disease and potential ways of risk reduction [3]. Studies of classical AD- and vascular pathologies have shown that these conditions are common in both older people with dementia and those without [4, 5]. These pathologies are known to start occurring in older brains in late midlife, and have gained increasing attention in light of consistent findings from studies in high-income countries showing a decline in the incidence of dementia [6] over time.

Subjective cognitive decline (SCD), i.e., the subjective perception of decline in memory or other cognitive domains in the absence of severe objective cognitive impairments or limitations in daily functioning, has been deemed a promising early risk marker for cognitive decline, MCI and AD, which is inexpensive, noninvasive and easy to obtain in regular healthcare settings [7, 8]. Presence of SCD was associated with a higher likelihood of MCI and dementia, shorter conversion time, and neuroimaging markers of AD in several memory-clinic and population-based studies [9–19]. More recently, the concept gained attention because focusing solely on MCI both identifies only a small proportion of individuals who develop dementia in the population, and due to mixed evidence from trials with limited impact on cognitive deterioration.

Subjective memory problems are highly common among older adults, especially when assessed using a single item, as applied in many epidemiological studies. Furthermore, SCD can also occur due to other conditions, e.g., depression or anxiety can enhance negative perceptions of one’s capabilities regarding memory or concentration [7, 20, 21]. This raises the question of the extent to which SCD reflects underlying cognitive decline or AD pathology. In order to enhance the utility of SCD as an early marker of the specific subtype of dementia, AD, further criteria have been suggested by an international expert consortium (SCD I working group), termed SCD Plus criteria:

- Subjective decline in memory, rather than other cognitive domains.
- Onset ≤ 5 years.
- Age at onset ≥ 60 years.
- Concerns about SCD.
- Perception of worse memory performance than others of the same age.

Further criteria include confirmation of cognitive decline by an informant, ApoE ε4 genotype, biomarker evidence for AD, persistence of SCD over time, and seeking medical help due to SCD [7]. SCD Plus criteria are subject to ongoing expansion and refinement [22].

While assessment of SCD and, therefore, availability of SCD Plus criteria varies largely across studies [3], certain studies found that a particular age at onset (≥ 60 years) increased the risk of conversion from SCD to dementia [11], predicted impaired cognitive performance [23] and amyloid positivity [23]. Some studies ([19, 24, 25] and [26]) found age-anchored SCD (i.e., memory decline compared to others of their own age) to be the most accurate predictor of objective cognitive performance. Informant-confirmed and age-anchored SCD notably increased the risk of progression to dementia in a population-based Swedish cohort [27]. Conversion rate to MCI was 18.9% in those who fulfilled all SCD Plus criteria, compared to 5.6% in subjects with only some cognitive concerns in a Spanish cohort study over a mean of 13 months (range: 10.7–22.4; [28]). Endorsement of SCD Plus features, particularly worries about SCD, was linked to higher amyloid beta and tau-burden in a recent meta-analysis of cohort studies, suggesting that SCD Plus might constitute an early marker of preclinical AD [25].

While evidence on (modifiable) risk and protective factors for objective cognitive decline and dementia has grown tremendously in the last two decades, far less is known about factors linked to the presence of SCD and if established risk factors for dementia, e.g., depression, diabetes mellitus, hearing loss, smoking, or low levels of education, are also linked to higher prevalence of SCD [29]. Many older adults with SCD seek medical help and advice [3], and with new amyloid antibody therapies becoming available in growing parts of the world, interest in early diagnosis of dementia and possible ways to reduce the risk of disease among older adults with SCD can be expected to increase. Therefore, knowledge of factors associated with SCD seems highly warranted.

Against this background, we aimed to (1) assess the prevalence of SCD Plus, using harmonized criteria, in a large sample comprising three population-based European cohorts of older adults; (2) investigate the links between sociodemographic, psychosocial, somatic, and lifestyle factors and SCD Plus.

## Methods

### Studies and participants

The present study analyzes data from three European population-based cohorts, i.e., the LIFE-Adult-Study (LIFE) from Germany; the English Longitudinal Study of Ageing (ELSA), and the Medical Research Council Cognitive Function and Ageing Study (CFAS) from the United Kingdom. We included participants aged ≥ 60 years, without a diagnosis of dementia at baseline, severe impairments in instrumental activities of daily living (IADL), or markedly impaired cognitive performance (≥ 1.5 SD below age-, sex-, and education-standardized norms).

The LIFE-Adult-Study aims to assess the prevalence and incidence of civilization diseases (e.g., depression, diabetes, stroke) and to investigate interactions between genetic and lifestyle factors in the occurrence of aforementioned diseases. The study comprises an age- and sex-stratified random sample of 10,000 community-dwelling adults at baseline, drawn from lists provided by the residents’ registration office of the city of Leipzig. Participants were invited by mail. Non-responders received one reminder letter and, where possible, were subsequently contacted by phone. Among individuals aged 40–79 years, the response rate was 31% [30]. After three participants withdrew their consent, the initial analytical sample included 9,997 individuals. Eligible participants were aged between 18 and 79 years at baseline. Baseline assessments were conducted at the Leipzig Research Center for Civilization Diseases (08/2011–11/2014), including physical examinations, psychometric tests, computer-assisted personal interviews, self-administered questionnaires, and clinical chemistry based on blood and urine samples. In-person and paper-based follow-up assessments were conducted between 10/2017 and 08/2021. Full details on LIFE are described elsewhere [30, 31].

ELSA is a longitudinal, multidisciplinary panel study conducted by University College London, comprising adults aged 50 to 100 at baseline (not sampling from care homes) aiming to be representative of the UK nationally, drawn from the Health Survey for England (HSE). The study assesses objective and subjective data on health and disability, biological markers of disease, socioeconomic circumstances, and psychosocial factors of ageing. At baseline (2002), *n* = 12,099 participants were interviewed in face-to-face visits by trained study nurses, using computer-assisted interviews and self-completed questionnaires. Individual response rate was 67%. There have been 9 further waves, with new panel recruitment at each phase. A proportion of the respondents have 9 follow-up interviews. Further details can be found elsewhere [32].

CFAS is a longitudinal study conducted by the University of Cambridge across five study centers in England and Wales (Cambridgeshire, Gwynedd, Newcastle upon Tyne, Nottingham, Oxford), investigating the prevalence and incidence of dementia and cognitive decline, depression, physical disability, neuropathology and molecular epidemiology in adults aged 65 years and older. Baseline interviews were conducted between 1991 and 1994 during face-to-face visits at participants’ homes. Response rate was about 80% [33]. A total of 13,004 participants completed the initial screening interview (1991–1994), which was used in the present study. Subsequent follow-ups were conducted in selected subgroups in 1997, 1999 and 2001, and all surviving participants were re-approached. In later phases, more intensive follow-up focused on specific subsamples, including participants expressing interest in brain donation. Further details on CFAS can be found elsewhere [33].

For CFAS and LIFE, baseline-data were used, while ELSA contributed data from wave two (two years after baseline; *n* = 9,432), since the questions required for SCD Plus were only asked from this wave onward.

### Assessment and harmonization of SCD Plus criteria

SCD Plus was operationalized based on available variables in each cohort, following the SCD Plus framework [7]. Due to differences in content of questionnaires, not all SCD Plus features were directly available in all three cohorts, therefore, proxy variables were used where necessary. This resulted in a harmonized approximation of the SCD Plus construct (see below). In LIFE and ELSA, four criteria included in SCD Plus were available: (1) self-reported decline in memory; (2) associated concern about declining memory performance; (3) onset of memory problems within the past five years; (4) confirmation of memory problems by an informant. Because ELSA lacks a direct item assessing concern about memory decline, poor self-rated memory (score ≥ 4 on a 1–5 scale) was used as a proxy for concern. The last criterion (informant confirmation) was not available in CFAS. All criteria were dichotomized as present or absent. Across cohorts, SCD Plus was defined as present if at least two of these available criteria were met. This harmonization approach enabled a comparable approximation of SCD Plus across studies. The full set of respective questions from each study is described in Supplementary Table 1. For ELSA, the item assessing comparison of memory to two years ago was used both to indicate subjective cognitive decline (worse memory) and to approximate recent onset.

### Demographics

We included information on participants’ sex, age at time of interview, and education in our analyses. Education was provided in years in full-time education in CFAS, while LIFE and ELSA provided categorical information on level of education. Information was harmonized into a categorical measure of education levels (low, intermediate, and high), based on the highest educational attainment in each cohort. In LIFE, this classification was derived from a composite measure of formal and vocational education (range 1.0–7.0), with predefined cut-offs (≤ 2.8 = low; >2.8 to < 4.55 = intermediate; ≥4.55 = high), corresponding to no or basic school education without vocational training, secondary education with vocational training, and higher education or university degree. In ELSA and CFAS, study-specific education variables (e.g., highest qualification or years of education) were mapped to this three-level classification to ensure comparability across cohorts. For details regarding harmonization, please see Supplementary Table 2. All studies provided information on marital status and the number of people living in the participant’s household.

### Covariates

Selection of factors potentially associated with SCD Plus was guided by current evidence regarding risk and protective factors for dementia, e.g., the latest report of the Lancet Commission on Dementia Prevention, Intervention and Care [34], describing 14 potentially modifiable risk factors for dementia: low levels of education, untreated hearing loss, untreated visual loss, hypertension, diabetes mellitus, obesity, high alcohol consumption, physical inactivity, traumatic brain injury, elevated levels of low-density lipoprotein (LDL) cholesterol, depression, smoking, social isolation, and exposure to air pollution.

Regarding SCD, several studies further reported associations between sleep problems and an elevated risk of SCD [29, 35, 36], which has been suggested as a candidate risk factor for dementia in the current report of the Lancet Commission [34]. Certain studies found links between SCD and personality traits, e.g. higher levels of neuroticism and lower levels of openness and conscientiousness [37, 38]. ApoE ε4 allele status was assessed as a non-modifiable factor, where available. Lastly, we assessed cognitive performance using standardized neuropsychiatric assessments conducted in each study. Information on operationalization and harmonization of all covariates is given in Supplementary Table 2.

### Inclusion and exclusion criteria

We included participants who were at least 60 years old at time of the interview and had answered the respective questions on SCD Plus criteria. In accordance with established frameworks [22, 39], participants with a diagnosis of dementia, severe IADL impairments, or cognitive performance > 1.5 SD below age-, sex- and education-specific norms for the respective cohort were excluded form analyses. In LIFE, mild and major neurocognitive disorder, according to DSM 5-criteria, were defined based on performance in neurocognitive tests (Trail Making Test A and B, Consortium to Establish a Registry for Alzheimer’s Disease (CERAD) Word List Memory, Verbal Fluency Test, CERAD Constructional Praxis Test, Reading the Mind in the Eyes-Test (revised version)), performance in activities of daily living (ADL; SIDAM (Structured Interview for the Diagnosis of Dementia of the Alzheimer Type, Multiinfarct-Dementia and Dementia of other Etiology)-ADL scale), as well as information on delirium (SIDAM delirium item) and further diagnoses (e.g., major depression, schizophrenia) possibly explaining cognitive deficits [40]. ELSA employed an operational case definition of dementia, based on self-reported physician diagnoses, complemented by information from the Informant Questionnaire on Cognitive Decline in the Elderly (IQCODE), assessing cognitive changes over time based on informant reports. Dementia diagnoses in CFAS were derived using the Automated Geriatric Examination for Computer Assisted Taxonomy (AGECAT) algorithm, based on the Geriatric Mental State Examination (GSM) [41]. AGECAT applies standardized algorithms to symptom data to generate syndrome-specific diagnostic levels. Individuals reaching an organicity AGECAT score ≥ O3 were classified as having dementia. Further details on exclusion criteria are provided in Supplementary Table 2.

### Statistical analyses

Sample characteristics for the total sample and individual studies are described using percentages (absolute numbers) or means and standard deviations, as appropriate. The prevalence of SCD Plus, using a harmonized operationalization, is reported for the total sample and per study, with 95% confidence intervals (CIs). Associated factors of SCD Plus are investigated as follows:

Firstly, associations of sociodemographic, psychosocial, somatic and lifestyle factors with SCD Plus were analyzed in the pooled dataset using generalized linear models (GLMs) with a Poisson-distribution, log link and robust standard errors to control for within-study correlation. This approach was chosen due to the expected non-rare prevalence of SCD Plus in order to provide stable estimates of prevalence ratios (PR) and avoid the risk of overestimation inherent to odds ratios (ORs) in outcomes with high prevalence. Sensitivity analyses were performed, excluding participants with depression or anxiety disorder (for operationalization of anxiety and depression see Supplementary Table 2). To further ensure robustness of results and directly account for between-study heterogeneity (one-stage approach; [42]), mixed-effects logistic regression models with study as a random intercept were conducted as additional sensitivity analyses.

Secondly, GLMs applying the same set of covariates were run separately in each cohort to examine study-specific associations.

Thirdly, to maximize the use of available information in each study, additional secondary analyses were performed per study, including covariates that were not assessed across all included cohorts (e.g., lifestyle factors or chronic conditions) to assess their individual associations with SCD Plus, controlling for age, sex, and level of education.

All analyses were conducted using Stata 19.0, with an alpha-level of 0.05 (two-tailed) indicating significance.

## Results

### Descriptive analyses

**Fig. 1:**
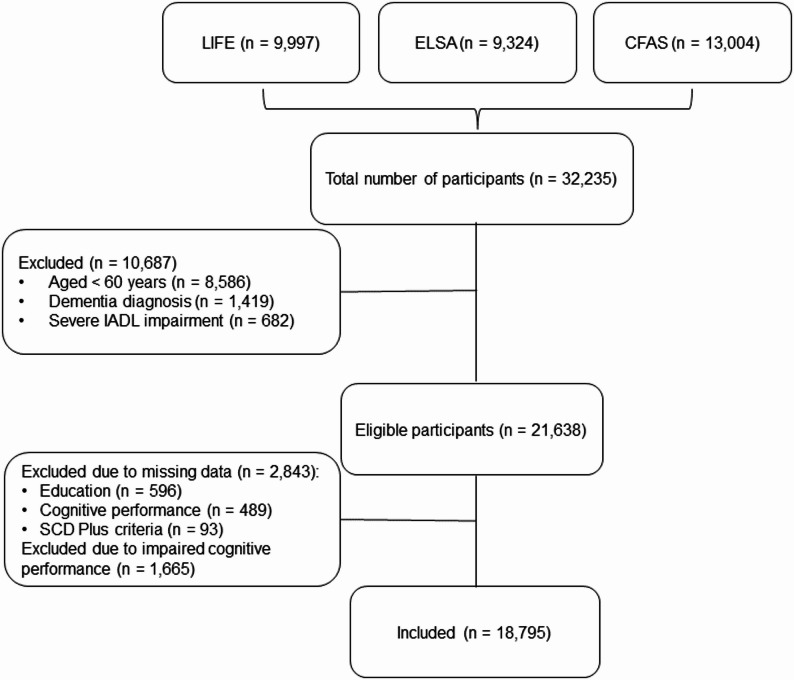
Flowchart of participants

Participant flow is described in Fig. 1. After exclusion of participants younger than 60 years at baseline (LIFE and ELSA only, as CFAS included participants aged ≥ 65 years at baseline) or with diagnosed dementia, severe IADL impairments, missing information on the harmonized SCD Plus construct, education or cognitive performance, or cognitive performance ≥ 1.5 SD below age-, sex- and education-specific norms, the analyzed sample included 18,795 observations (3,717, 4,656 and 10,422 from LIFE, ELSA and CFAS, respectively).

Table 1 describes the characteristics of participants included in the present study.

**Table 1:** Sample characteristics of the total sample and by cohort

Variable	Overall (*n* = 18,795)	LIFE (*n* = 3,717)	ELSA (*n* = 4,656)	CFAS (*n* = 10,422)
	Mean (SD) / % (*n*)			
Age, years; range	72.1 (6.8); 60.0–99.0	68.6 (5.2); 60-80.9	70.5 (7.5); 60.0–90.0	74.1 (6.2); 64.0–99.0
Women	55.5 (10,422)	51.7 (1,921)	53.7 (2,498)	57.6 (6,003)
Education				
Low	46.7 (8,779)	1.3 (47)	51.7 (2,406)	60.7 (6,326)
Intermediate	35.7 (6,709)	55.9 (2,079)	36.1 (1,6823)	28.3 (2,948)
High	17.6 (3,307)	42.8 (1,591)	12.2 (568)	17.6 (3,307)
Married/partnership (ref.: single/divorced/widowed)	61.9 (11,637)	77.9 (2,895)	66.1 (3,075)	54.4 (5,667)
Living alone (ref.: multi-person household)	33.0 (6,178)	23.8 (882)	29.8 (1,387)	37.7 (3,909)
Depression	14.9 (2,675)	11.6 (427)	21.5 (1,001)	13.0 (1,247)
Anxiety	5.5 (942)	4.1 (142)	1.2 (52)	8.1 (748)
Hypertension	43.3 (8,093)	70.7 (2,557)	45.6 (2,124)	32.8 (3,412)
Diabetes mellitus	9.4 (1,763)	22.1 (809)	8.8 (409)	5.2 (545)
Heart disease	19.6 (3,625)	26.7 (905)	19.9 (926)	17.2 (1,794)
Parkinson’s disease	0.5 (99)	0.4 (15)	0.6 (26)	0.6 (58)
History of stroke	4.4 (820)	2.9 (105)	5.0 (232)	4.6 (483)
Smoking	15.8 (2,933)	10.5 (373)	12.5 (583)	19.0 (1,977)
Cognitive performance (composite z-score); Mean (SD)	0.1 (0.7)	-0.1 (0.6)	0.0 (0.8)	0.3 (0.6)

Values are presented as mean (SD) or percentage (*n*). *CFAS* Cognitive Function and Ageing Study, *ELSA* English Longitudinal Study of Ageing, LIFE: LIFE-Adult-Study; ref.: reference. Cognitive performance was assessed using a study-specific composite z-score. Percentages are based on available data for each variable; denominators may vary across cohorts and variables due to missing values. Missing values (*n*) per study: partnership (ELSA: 1, CFAS: 1), living alone (LIFE: 3, CFAS: 64), depression (LIFE: 26, ELSA: 6, CFAS: 797), anxiety (LIFE: 210, ELSA: 242, CFAS: 1,154), hypertension (LIFE: 98, CFAS: 7), smoking (LIFE: 173, ELSA: 2, CFAS: 3), diabetes (LIFE: 48, CFAS: 4), heart disease (LIFE: 324, CFAS: 6), stroke (LIFE: 42, CFAS: 2), Parkinson’s disease (LIFE: 204, CFAS: 4)

The overall prevalence of SCD Plus, based on the harmonized operationalization, was 37.9% (95% CI: 37.3, 38.6) in the total sample, with 53.7% (95% CI: 52.1, 55.3), 35.7% (95% CI: 34.4, 37.1) and 33.3% (95% CI: 32.4, 34.2) in LIFE, ELSA and CFAS, respectively (*n* = 1,996, 1,664, 3,472). Prevalence of the harmonized SCD Plus construct did not differ by sex (*p* = .064) in the total sample, but was higher in participants with an intermediate or higher level of education compared to those with a low level of education (*p* < .001, respectively). Prevalence of SCD Plus did not differ by age in pooled analyses (linear; *p* = .488; age group, 60–69, 70–79, ≥ 80: *p* = .142).

When excluding participants with depression (*n* = 2,675) or anxiety disorder (*n* = 942), the prevalence of the harmonized SCD Plus-construct was 36.4% in the total sample (95% CI: 35.6, 37.2). Values for LIFE, ELSA, and CFAS were 52.1% (95% CI: 50.4, 53.8), 33.0% (95% CI: 31.5, 34.5), and 31.9% (95% CI: 30.9, 32.9), respectively (not tabulated).

### Associated factors of SCD Plus

Table 2 describes associations of sociodemographic, psychosocial, somatic, and lifestyle factors with SCD Plus in the total sample, using a generalized linear model with Poisson-distribution, log link, and robust standard errors. Variance inflation factors (VIF) were calculated, with all values ≤ 4.39 (“married/living in a partnership; 4.27: “living alone”), indicating no signs of critical multicollinearity.

Prevalence of SCD Plus, based on the harmonized operationalization, was not linked to age or sex in pooled analyses, while a high level of education (PR: 1.35, 95% CI: 1.06, 1.72) was associated with higher probability of SCD Plus. Depression (PR: 1.28, 95% CI: 1.20, 1.36), anxiety (PR: 1.34, 95% CI: 1.17, 1.54), hypertension (PR: 1.07, 95% CI: 1.03, 1.10), diabetes (PR: 1.09, 95% CI: 1.01, 1.12), heart disease (PR: 1.10, 95% CI: 1.03, 1.19), Parkinson’s disease (PR: 1.21, 95% CI: 1.11, 1.31), and history of stroke (PR: 1.10, 95% CI: 1.04, 1.17) were linked to higher probability of SCD Plus. Smoking and better cognitive performance were inversely linked to SCD Plus (PR: 0.87, 95% CI: 0.80, 0.95; PR: 0.90, 95% CI: 0.85, 0.95, respectively).

**Table 2:** Multivariable associations with SCD Plus in the total sample (Poisson regression)

Variable	PR	95% CI	*p*
Age	1.00	1.00, 1.01	.595
Female sex	1.06	0.99, 1.12	.083
Education intermediate (ref.: low)	1.23	0.96, 1.57	.107
Education high	1.35	1.06, 1.72	**.013**
Married/partnership (ref.: single/divorced/widowed)	1.12	0.98, 1.28	.106
Living alone (ref.: multi-person household)	0.99	0.98, 1.01	.508
Depression	1.28	1.20, 1.36	**< .001**
Anxiety	1.34	1.17, 1.54	**< .001**
Hypertension	1.07	1.03, 1.10	**< .001**
Diabetes mellitus	1.09	1. 01, 1.12	**< .001**
Heart disease	1.10	1.03, 1.19	**.009**
Parkinson’s disease	1.21	1.11, 1.31	**< .001**
History of stroke	1.10	1.04, 1.17	**.001**
Smoking	0.87	0.80, 0.95	**.002**
Cognitive performance (composite z-score)	0.90	0.85, 0.95	**< .001**

*CI* confidence interval, *PR* prevalence ratio, *ref* reference. All variables were entered simultaneously into the model (generalized linear model with a Poisson distribution, log link, and robust standard errors)

Boldface indicates statistical significance at the p < 0.05 level

Sensitivity analyses, excluding participants with anxiety disorder (*n* = 942) or depression (*n* = 2,675), revealed highly consistent results (Supplementary Table 3). A high level of education, living alone, diagnoses of hypertension, diabetes, heart disease, Parkinson’s disease, and a history of stroke were linked to higher probability of SCD Plus in the respective model, while smoking and better cognitive performance were inversely associated with SCD Plus in pooled analyses.

To directly account for between-study heterogeneity and assess the robustness of our findings, further sensitivity analyses were conducted applying a mixed-effects logistic regression model with a study-level random intercept, resulting in highly comparable findings (Supplementary Table 4). Estimated variance of the study-level random intercept was 0.10 (95% CI: 0.02, 0.53), with a corresponding ICC of 0.03, indicating modest heterogeneity in the prevalence of SCD Plus, based on the harmonized operationalization, across the three cohorts.

Further sensitivity analyses applying a stricter, fully harmonized definition of SCD Plus (requiring both: 1) subjective decline in memory and: 2) worry about memory problems) were conducted to account for variations in availability and operationalization SCD Plus criteria across cohorts. Results were largely consistent with primary analyses (Table 2), although some associations were attenuated and no longer significant when applying the stricter SCD Plus operationalization, which might reflect reduced statistical power due to the more restrictive definition (hypertension: PR = 1.07, 95% CI: 0.95, 1.21; *p* = .280; Parkinson’s disease: PR = 1.43, 95% CI: 0.96, 2.15; *p* = .082; smoking: PR = 0.93, 95% CI: 0.84, 1.03; *p* = .177; cognitive performance: PR = 0.84, 95% CI: 0.70, 1.01; *p* = .061; see Supplementary Table 5).

In cohort-specific analyses (Table 3), using the same set of covariates, several associations were not consistent with results from pooled analyses, indicating heterogeneity between cohorts. Higher age was linked to a higher probability of SCD Plus in LIFE (PR: 1.01, 95% CI: 1.00, 1.02) and CFAS (PR: 1.01, 95% CI: 1.01, 1.02). An intermediate (PR: 1.26, 95% CI: 1.12, 1.41) and high level of education (PR: 1.42, 95% CI: 1.20, 1.67) was linked to SCD Plus in ELSA, but not in LIFE or CFAS. ELSA-participants who were married or living in a partnership had higher probabilities of SCD Plus (PR: 1.34, 95% CI: 1.04; 1.72). Depression was linked to higher likelihood of SCD Plus in all cohorts (LIFE: PR: 1.23, 95% CI: 1.06, 1.43; ELSA: PR: 1.35, 95% CI: 1.19, 1.52; CFAS: PR: 1.41, 95% CI: 1.17, 1.69), while anxiety was linked to SCD Plus in ELSA (PR: 1.53, 95% CI: 1.06, 2.20) and CFAS (PR: 1.31, 95% CI: 1.02, 1.69). Heart disease was associated with higher probability of SCD Plus in CFAS (PR: 1.16, 95% CI: 1.05, 1.27), while no further associations with somatic conditions were detected. In CFAS, smoking was inversely linked to SCD Plus (PR: 0.86, 95% CI: 0.77, 0.95), while better cognitive performance was linked to lower probability of SCD Plus in ELSA (PR: 0.92, 95% CI: 0.86, 1.00).

**Table 3:** Factors associated with SCD Plus by cohort (multivariable Poisson regression models)

Variable	LIFE			ELSA			CFAS		
	PR	95% CI	*p*	PR	95% CI	*p*	PR	95% CI	*p*
Age	1.01	1.00, 1.02	**.022**	1.00	0.99, 1.01	.685	1.01	1.01, 1.02	**.001**
Female sex	1.09	0.98, 1.22	.097	1.02	0.92, 1.13	.705	1.03	0.95, 1.12	.435
Education intermediate (ref.: low)	0.84	0.55, 1.28	.416	1.26	1.12, 1.41	**< .001**	1.00	0.92, 1.09	.979
Education high	0.82	0.54, 1.26	.369	1.42	1.20, 1.67	**< .001**	1.03	0.91, 1.17	.608
Married/partnership (ref.: single/divorced/widowed)	0.95	0.70, 1.29	.745	1.34	1.04, 1.72	**.022**	1.01	0.89, 1.14	.906
Living alone (ref.: multi-person household)	0.85	0.63, 1.14	.284	1.04	0.81, 1.35	.746	0.98	0.86, 1.11	.716
Depression	1.23	1.06, 1.43	**.007**	1.35	1.19, 1.52	**< .001**	1.41	1.17, 1.69	**< .001**
Anxiety	1.19	0.94, 1.50	.150	1.53	1.06, 2.20	**.023**	1.31	1.02, 1.69	**.038**
Hypertension	0.96	0.86, 1.08	.525	1.04	0.94, 1.15	.448	1.02	0.95, 1.11	.557
Diabetes mellitus	0.99	0.88, 1.12	.853	1.10	0.93, 1.30	.269	1.02	0.86, 1.19	.854
Heart disease	1.06	0.94, 1.18	.347	1.01	0.89, 1.15	.837	1.16	1.05, 1.27	.**003**
Parkinson’s disease	1.33	0.71, 2.48	.369	1.09	0.60, 1.97	.780	1.24	0.79, 1.94	.359
History of stroke	1.07	0.79, 1.44	.665	1.19	0.96, 1.47	.115	1.14	0.96, 1.34	.132
Smoking	1.00	0.84, 1.18	.966	0.91	0.78, 1.08	.283	0.86	0.77, 0.95	**.004**
Cognitive performance (composite z-score)	0.98	0.90, 1.06	.636	0.92	0.86, 1.00	.**041**	0.96	0.90, 1.03	.235

*CI* confidence interval, *PR* prevalence ratio, *ref* reference; generalized linear models with Poisson-distribution and log-link function. Each column represents a separate multivariable model per cohort, including the same set of covariates. *CFAS* Cognitive Function and Ageing Study, *LIFE*  LIFE-Adult-Study, *ELSA* English Longitudinal Study of Ageing

Boldface indicates statistical significance at the p < 0.05 level

Individual associations of sociodemographic, psychosocial, lifestyle, or somatic factors with SCD Plus in each cohort (adjusted for age, sex, and level of education, respectively) are described in Table 4.

Being married/living in a partnership was individually linked to SCD Plus in ELSA, whereas the likelihood of SCD Plus was lower among ELSA-participants who were living alone. Depression and anxiety were individually associated with SCD Plus in all cohorts. In ELSA, a history of stroke was linked to a higher probability of SCD Plus, while better cognitive performance was linked to a lower likelihood of SCD Plus. Among CFAS-participants, heart disease and history of stroke were linked to higher probability of SCD Plus, while smoking was inversely linked to SCD Plus. Among factors with limited availability across cohorts, high levels of physical activity (ref.: low levels of physical activity) were linked to a lower likelihood of SCD Plus in ELSA but not in LIFE. Sleep problems were linked to SCD Plus both in LIFE and CFAS. Hearing problems were associated with a higher likelihood of SCD Plus in both ELSA and CFAS. Neither higher alcohol consumption, level of LDL cholesterol, ApoE ε4 allele status, nor exposure to air pollution was linked to SCD Plus in the respective cohorts with available data. In LIFE, higher levels of neuroticism were associated with SCD Plus, while greater openness, agreeableness, and conscientiousness were inversely linked to SCD Plus. In CFAS, higher probability of SCD Plus was associated with thyroid disease.

**Table 4:** Age-, sex- and education-adjusted associations with SCD Plus by cohort

Variable	LIFE			ELSA			CFAS		
	PR	95% CI	*p*	PR	95% CI	*p*	PR	95% CI	*p*
*Core set of factors*,* available across studies*									
Married/partnership (ref.: single/divorced/widowed)	1.05	0.94, 1.17	.387	1.24	1.11, 1.39	**< .001**	1.00	0939, 1.08	.944
Living alone (ref.: multi-person household)	0.93	0.84, 1.04	.210	0.84	0.75, 0.94	**.003**	1.01	0.94, 1.08	.860
Depression	1.23	1.08, 1.40	**.002**	1.41	1.26, 1.58	**< .001**	1.29	1.17, 1.42	**< .001**
Anxiety	1.31	1.07, 1.61	**.009**	1.66	1.16, 2.39	**.006**	1.25	1.11, 1.41	**< .001**
Hypertension	0.96	0.87, 1.06	.449	1.09	0.99, 1.20	.093	1.06	0.99, 1.14	.093
Diabetes mellitus	0.97	0.87, 1.08	.523	1.16	0.99, 1.36	.072	1.04	0.90, 1.20	.613
Heart disease	1.07	0.96, 1.19	.209	1.10	0.98, 1.24	.117	1.16	1.07, 1.27	**.001**
Parkinson’s disease	1.39	0.77, 2.52	.276	1.16	0.64, 2.10	.623	1.33	0.90, 1.95	.150
History of stroke	1.09	0.85, 1.41	.481	1.30	1.07, 1.59	**.009**	1.24	1.08, 1.43	.**003**
Smoking	0.97	0.84, 1.14	.743	0.94	0.80, 1.09	.406	0899	0.81, 0.97	.**010**
Cognitive performance (composite z-score)	0.97	0.90, 1.04	.424	0.90	0.84, 0.97	**.004**	0.95	0.89, 1.00	.070

	**LIFE**			**ELSA**			**CFAS**		
*Additional factors with limited availability across studies*	PR	95% CI	*p*	PR	95% CI	*p*	PR	95% CI	*p*
Moderate physical activity (ref.: low)	1.05	0.87, 1.26	.874	0.92	0.81, 1.03	.152	n.a.		
High physical activity	1.01	0.85, 1.21	.850	0.86	0.74, 0.99	**.039**	n.a.		
Alcohol consumption (g/day)	1.00	1.00, 1.01	.095	1.00	1.00, 1.00	.616	n.a.		
LDL cholesterol	1.00	0.95, 1.05	.969	0.95	0.89, 1.01	.083	n.a.		
Sleep problems	1.05	1.03, 1.07	**< .001**	n.a.			1.30	1.21, 1.39	**<.001**
Hearing problems	n.a.			1.48	1.33, 1.65	**<.001**	1.32	1.22, 1.43	< .**001**
ApoE ε4 carrier	1.02	0.92, 1.13	.732	n.a.			1.12	0.86, 1.42	.344
Thyroid disease	1.07	0.97, 1.18	.103	n.a.			1.16	1.03, 1.31	.**012**
Air pollution (PM₂.₅ (µg/m³), 5-year mean	1.00	0.92, 1.09	.993	n.a.			n.a.		
Neuroticism	1.11	1.06, 1.16	**< .001**	n.a			n.a		
Extraversion	1.01	0.97, 1.06	.592	n.a			n.a		
Openness	0.94	0.89, 0.99	**.020**	n.a			n.a		
Agreeableness	0.95	0.91, 1.00	**.046**	n.a			n.a		
Conscientiousness	0.92	0.87, 0.98	**.007**	n.a			n.a		

*ApoE* Apolipoprotein E, *CI* confidence interval, *LDL* low-density lipoprotein, *PR* prevalence ratio, *ref* reference. All models are adjusted for age, sex, and level of education. Each row represents a separate model, including one exposure variable and the three covariates

Boldface indicates statistical significance at the p < 0.05 level

## Discussion

We analyzed the prevalence of an updated, expert-based concept of subjective cognitive decline (SCD Plus) in older adults from three large, population-based European cohorts, applying a harmonized operationalization approach. Further, we reported associations between sociodemographic, psychosocial, somatic, and lifestyle-related factors and the harmonized SCD Plus criteria both in the total sample and within each cohort. Overall prevalence of SCD Plus, as operationalized in our study, was 37.9%, ranging from 33.3% to 53.7% across studies.

### Prevalence of SCD Plus

The observed prevalence rate in our study falls within the broad range of SCD prevalence reported in previous population-based studies. For instance, Röhr and colleagues assessed the prevalence of SCD across 16 cohorts of the Cohort Studies of Memory in an International Consortium (COSMIC), reporting an overall prevalence of 23.8%, ranging from 6.1 to 52.7% across studies [43]. More recent studies, e.g., from Wales or China, reported prevalences of SCD of 51.5% [44], 41% [45] and 42% [29], while other cohort studies found rates of SCD below 20% [36, 46, 47]. Prevalence rates observed in the current study are further in line with findings from a recent meta-analysis of nine cohort studies, using SCD Plus criteria [25], as well as a study on the prevalence of SCD Plus in China (43.8% [35]). It should be considered, however, that assessment of SCD criteria, as well as the age ranges of study participants, differed across studies, complicating direct comparison.

The prevalence of SCD Plus within the cohorts included in the present study, based on a harmonized operationalization, was highest in LIFE (53.7%) and lowest for CFAS (33.3%). These differences might partially be explained by differences in age, sex, educational distributions, wording, and response options of respective items, as well as different timepoints of assessment across cohorts. In line with our findings, Röhr and colleagues [43] reported a higher prevalence of SCD in cohorts conducted in more recent decades. Further, differences in prevalence of somatic comorbidities may have contributed to the higher prevalence of SCD Plus observed in LIFE. Participants of this cohort showed a higher burden of hypertension, diabetes, and heart disease, which were associated with SCD Plus in our study. Further, the LIFE-Adult cohort is composed of comparatively health-conscious and socioeconomically advantaged individuals: Engel and colleagues reported that participants were more often highly educated, healthier, and less likely to smoke, compared to the general population and non-participants [30]. This could likely suggest higher health awareness in this cohort, which may increase the likelihood of reporting subjective cognitive concerns, despite slightly lower mean age in LIFE-participants than in CFAS or ELSA. However, sensitivity analyses applying a study-level random intercept indicated only modest heterogeneity in prevalence of SCD Plus, as operationalized in this study, between cohorts, therefore, we are confident that respective estimates can nonetheless be interpreted as meaningful.

### Associated factors of SCD Plus

Regarding factors associated with SCD Plus, higher levels of education, depression, anxiety, as well as somatic comorbidities (hypertension, diabetes, heart disease, Parkinson’s disease, history of stroke) were linked to a higher likelihood of SCD Plus in the total sample (Table 2). Smoking, on the other hand, and better cognitive performance were inversely related to SCD Plus.

Findings regarding sociodemographic differences between older adults with and without SCD are mixed. While Röhr and colleagues [43] reported a higher prevalence of SCD in men and those with lower education, other studies found no sociodemographic differences regarding the prevalence of SCD [35, 37, 48, 49], or a higher prevalence of SCD in women [29, 50]. Interestingly, we found a higher likelihood of SCD Plus in participants with higher levels of education, although mainly driven by ELSA-participants, without consistent associations across cohorts. While respective associations have also been reported in other studies [50], it stands in contrast to the well-established protective effects of education against dementia. This might reflect the subjective nature of SCD: Individuals with higher levels of education might be more aware of subtle cognitive changes and more likely to report early signs of perceived decline, potentially due to higher health literacy [51, 52] and sensitivity to early symptoms. Further, differences in reporting behavior across educational groups might have contributed to the observed association.

In line with previous studies, SCD Plus was linked to diagnoses of heart disease [35, 50], diabetes [35, 36, 46, 53], Parkinson’s disease [54], history of stroke [55], and hypertension [46], suggesting that these established risk factors for dementia might also be associated with SCD. Depression and anxiety were consistently linked to SCD Plus, even after simultaneous adjustment for multiple sociodemographic, somatic, and psychosocial factors, as has been reported in numerous other studies [36, 37, 46, 49]. Since both depression and anxiety are established risk factors for objective cognitive decline and dementia, these findings underline the importance of assessing affective symptoms when evaluating subjective concerns about memory, and of considering the possibility of bidirectional or mutually reinforcing pathways. Sensitivity analyses, excluding participants with depression and/or anxiety, only marginally changed results.

Several cardiometabolic comorbidities linked to SCD Plus, e.g., hypertension, diabetes, or history of heart disease, were more prevalent among LIFE-participants. However, this does not necessarily indicate a geographically increased disease burden. Estimates from population-based studies suggest that hypertension affects up to two thirds of older adults Germany [56, 57], and diabetes prevalence also increases with age [58, 59]. Therefore, the estimates observed in LIFE in our study are within the expected range for older population-based samples, suggesting that differences between cohorts might more likely indicate variations in age structure, study design, and assessment procedures. Further, cohort effects may contribute to the observed differences, as LIFE was conducted more recently than CFAS and ELSA, i.e., changes in diagnostic practices and increased awareness over time may have led to higher detection rates of cardiometabolic conditions.

Better cognitive performance was linked to lower probability of SCD Plus, even when controlling for symptoms of depression and anxiety or when participants with depression/anxiety were excluded from analyses. While several studies reported similar associations [50, 53, 60–62], others found no association between objective cognitive performance and SCD Plus [37, 48, 49, 63, 64]. Our findings therefore suggest that SCD Plus might be an indicator of objectively measurable lower cognitive performance in population-based samples. Inconsistencies regarding associations of SCD and cognitive performance between studies might, at least partially, be due to different assessments of SCD (Plus) features, as well as differences in age ranges and sampling strategies. Standardized instruments assessing SCD Plus features that provide valid estimates across different settings and groups of participants are highly warranted.

The inverse association between smoking and reporting SCD Plus was unexpected and should be interpreted with caution. It should be noted that this association was primarily driven from CFAS-participants, while no significant association between current smoking and SCD Plus was observed in ELSA or LIFE, suggesting that this finding could reflect cohort-specific factors rather than a generalizable association. For one, the association might be explained by selective survival of healthier smokers, i.e., less healthy smokers are less likely to survive into older age. Smoking-related morbidity and health problems may further reduce the likelihood of study participation, which may have resulted in a comparatively healthier and more functional subgroup of smokers in this study [65]. Further, SCD is inherently subjective and may be influenced by differences in symptom perception and reporting behavior. Smokers might be less likely to notice or report subtle cognitive changes, or respective concerns might be overshadowed by competing health concerns, e.g., breathing difficulties or problems regarding mobility in smokers. Similar associations have been found in other studies examining SCD [50]. While certain trials in samples with unimpaired cognition or MCI suggest short-term positive effects of (transdermal) nicotine on cognitive performance, e.g., attention and memory [66, 67], these findings should not be translated directly to smoking, which is consistently associated with an increased risk of cognitive decline and dementia [34]. In conclusion, the inverse association between smoking and SCD Plus features is likely due to methodological and cohort-specific factors, rather than suggesting a true protective effect. When investigating study-specific results from the multivariate models, older age was linked to SCD Plus in LIFE and CFAS, while higher education was associated with SCD Plus in ELSA. Depression was linked to a higher probability of SCD Plus in all cohorts, while an association with anxiety remained in ELSA and CFAS when controlling for somatic and psychosocial factors. Among somatic comorbidities, only the association of heart disease with higher probability of SCD Plus remained significant in fully adjusted models for CFAS. Associations with heart disease and stroke were found in CFAS when these factors were investigated individually, while history of stroke was individually linked to higher probability of SCD Plus in ELSA (controlling for age, sex, and education). Better cognitive performance was inversely linked to SCD Plus in ELSA and CFAS, while an inverse link between smoking and probability of SCD Plus was found in CFAS only. Differences between pooled and cohort-specific analyses might be due to increased power in the total sample (*n* = 18,795), allowing detection of associations between SCD Plus with conditions with a lower frequency and comparatively small prevalence ratios in our sample, e.g., Parkinson’s disease. While higher age was positively associated with probability of SCD Plus in LIFE and CFAS, no such association was observed in ELSA, and no respective link was detected in the pooled model. This suggests that the association between age and SCD Plus might be cohort-specific rather than universal. Differences in age distributions, baseline prevalence of SCD Plus, and study characteristics likely attenuated a global association when cohorts were pooled in one dataset.

In ELSA-participants, living alone was linked to a lower likelihood of SCD Plus, while those who were married/living in a partnership had higher odds. This might partly reflect the subjective nature of SCD Plus, with individuals living with others possibly receiving more feedback on cognitive performance memory problems, and thus might report more concerns. Further, the definition of SCD Plus in ELSA included an informant-based criterion, which is more likely to be fulfilled when people receive regular feedback from close ones, i.e., those living alone might therefore be less likely to fulfill this criterion. Since this finding was not observed in the other cohorts, however, it might likely reflect cohort-specific and methodological factors rather than suggest causal effects.

Regarding the association of higher levels of education and SCD Plus, respective associations were only found for ELSA in cohort-specific analyses. As the pooled analysis used educational variation across cohorts, the distribution of education levels was broader than in single studies, likely explaining the significant association of education with SCD Plus in the total sample, but not in LIFE or CFAS. Results from the pooled analyses likely provide the most robust and generalizable estimates, whereas analyses per study highlight the role of contextual, demographic, and measurement differences for the observed associations with SCD Plus. Respective differences are expected in harmonized multi-cohort analyses. While pooled models provide greater statistical power and result in more conservative estimates, study-specific analyses reveal which associations rely on population structure, assessment instruments, or cohort context.

The above paragraphs underline that several associations identified in the pooled analyses were not consistent across cohorts. As demonstrated in cohort-specific analyses (Table 3), statistically significant findings were largely driven by ELSA-participants, while results in CFAS and LIFE were weaker and, in few cases, differed in direction. Although sensitivity analyses suggested only modest heterogeneity between cohorts, this may reflect differences in measurement, population characteristics, as well as variation in the operationalization of SCD Plus across LIFE, ELSA and CFAS and highlights the need for cautious interpretation of pooled results. These findings underline the importance of examining cohort-specific findings when applying SCD Plus criteria across heterogeneous population-based samples.

Among factors with limited availability across cohorts, sleep problems emerged as robust correlates of SCD Plus in both LIFE and CFAS, corroborating findings from other studies [29, 35, 36, 50]. Hearing problems were linked to SCD Plus in ELSA and CFAS, while higher levels of physical activity reduced probability of SCD Plus in participants of ELSA, similar to findings from Voyer and colleagues [36]. Neither alcohol consumption, nor levels of LDL cholesterol were found to be associated with SCD Plus, while thyroid disease was linked to higher probability of SCD Plus, corroborating findings from Wen and colleagues [29]. LIFE was the only study to provide data on exposure to air pollution, however, no association was detected between this environmental risk factor for dementia and SCD Plus in the current study. Individuals with higher levels of neuroticism more often reported SCD Plus in LIFE, while openness, agreeableness and conscientiousness were inversely associated with SCD Plus. Similar findings were reported in the Sydney memory and Ageing Study [37], while lower values of neuroticism were reported in individuals reporting SCD in the Spanish Fundació ACE Health Brain Initiative (FACEHBI) study [68]. No association with ApoE ε4 genotype was detected, consistent with findings from other studies [27, 69], suggesting that ApoE ε4 genotype might be linked to objective rather than subjective cognitive performance.

### Strengths and limitations

Our study assessed prevalence and associated factors of SCD Plus criteria, using a harmonized operationalization, in a large, population-based sample, comprising more than 18,000 older adults from three well-characterized European cohorts. By applying a harmonized measure of established SCD Plus criteria and following expert working group recommendations, our findings go beyond studies that rely solely on a single-item definition of subjective memory problems. We implemented a systematic harmonization strategy for sociodemographic, psychosocial, somatic, and lifestyle variables, allowing us to examine a broad range of potential correlates of SCD Plus in both pooled and cohort-specific analyses. Pooling data from comparable cohorts, using harmonized criteria for SCD Plus and associated factors, allows identification of patterns that might otherwise be overlooked when analyzing single studies, e.g., due to a lack of statistical power. Using both pooled and study-specific analyses provides complementary insights, i.e., while pooled models offer generalizable estimates across cohorts, study-specific findings illustrate how associations vary by population structure, measurement properties, and other cohort-specific factors. Our findings may be of interest to both future research and clinical practice for identifying groups with a high probability of subjective memory problems.

Certain limitations need to be mentioned when interpreting our findings. A key limitation lies in the operationalization of SCD Plus across cohorts. Although we aimed to follow the SCD- framework as closely as possible, differences in available variables required the use of proxy indicators, and not all features of SCD Plus could be captured equivalently in the three cohorts. This implies that our SCD Plus construct might not be directly comparable across cohorts, and differences in prevalences and associated factors might in part reflect measurement differences instead of true variation across cohorts. A large number of observations had to be excluded due to missing information in single items, and not all participants in the three studies answered all potentially available questions regarding further SCD Plus features (e.g., comparison of memory problems to others the same age, which has been found a criterion particularly predictive of objective cognitive decline [24, 26, 27]), which may have provided a more comprehensive understanding of the prevalence of specific SCD Plus criteria. Due to the limited availability of additional SCD Plus features across included studies, we relied on a dichotomous operationalization. While this approach still comprises core features of SCD Plus, more comprehensive assessments would have allowed differentiation among degrees of SCD Plus severity, e.g., by the number of fulfilled criteria [3, 16]. Variability in assessments across studies poses a major challenge to research on SCD in general [70], and is especially heterogeneous in studies using community-based samples [3]. In the present study, we approximated the criterion of concern regarding memory problems in data from ELSA, using data from a rating scale of perceived memory, and there were differences in the cognitive performance tests used across studies. Therefore, differential measurement error could attenuate or inflate cohort-specific associations. What is more, the availability of data on factors potentially linked to SCD Plus varied across studies, i.e., certain associations could only be investigated in cohort-specific studies. As the cohorts included in the present study were conducted in different calendar periods, ranging from the early 1990s (CFAS) to the 2010s (LIFE), diagnostic practices and thresholds for certain conditions included in our analyses (e.g., diabetes or hypertension) may have varied slightly over time, posing challenges to pooling of respective data. Moreover, secular changes in health awareness, behaviors (e.g., declining prevalences of smoking; [71, 72]) and public understanding of cognitive impairment, driven by public health campaigns, national dementia strategies, advocacy of patient groups and broader education effort [73, 74] might influence both disease prevalence and subjective reporting of memory problems. Therefore, pooled analyses should be interpreted with caution, and alongside cohort-specific findings reported in our study. Further, as is common in population-based ageing studies, some degree of selection bias cannot be ruled out, as participants are often, on average, healthier than non-responders. Lastly, the cross-sectional nature of the present study does not allow for conclusions regarding causality of associated factors with SCD Plus.

## Conclusion

Given the absence of curative treatment options for AD, subjective cognitive decline, assessed using expert-based criteria (SCD Plus), constitutes a promising early risk marker for cognitive decline and dementia. In this large, population-based multi-cohort study, approximately 40% of older adults fulfilled criteria of SCD Plus, despite the absence of objective cognitive impairment. Several established risk factors for dementia were also linked to SCD Plus, e.g., depression, hypertension, heart disease, or diabetes, supporting its relevance for early risk assessment in community settings. Combining pooled and cohort-specific analyses allowed to identify both generalizable patterns and variations between cohorts, thereby highlighting the importance of considering contextual and measurement differences when investigating SCD Plus across populations. Our findings underscore the potential of SCD Plus as a potentially accessible marker to identify individuals at increased risk in clinical and public health settings. Further, they emphasize the need for more standardized assessments of SCD Plus features to ensure comparability across studies. Longitudinal studies applying SCD Plus criteria and simultaneously assessing respective associated factors will improve our understanding of whether these factors increase the risk of future cognitive decline and dementia in subjects with SCD Plus and to clarify the role of SCD Plus in risk stratification and early intervention strategies, taking into account regional, cultural, and temporal differences in the assessment and reporting of subjective cognitive decline.

## Supplementary Information

Supplementary Material 1.

## Data Availability

Due to privacy protection, restrictions apply to the availability of the data. Data from the LIFE-Adult-Study are available to researchers who submit a written proposal, including objectives, measures, names of all researchers involved, and how results and newly generated data will be returned for further use. Inquiries should be submitted to [info-life@lists.uni-leipzig.de]. Data from CFAS and ELSA used in this study are available to researchers who submit a sound proposal to the Dementias Platform UK (DPUK) Data Portal ([https://portal.dementiasplatform.uk/]).

## Abbreviations

AD: Alzheimer’s Disease
ADL: Activities of Daily Living
ApoE: Apolipoprotein E
CERAD: Consortium to Establish a Registry for Alzheimer’s Disease
CFAS: Cognitive Function and Ageing Study
COSMIC: Cohort Studies of Memory in an International Consortium
ELSA: English Longitudinal Study of Ageing
GLM: Generalized Linear Model
HSE: Health Survey for England
IADL: Instrumental Activities of Daily Living
LDL: Low-density Lipoprotein
LIFE: Life-Adult-Study
MCI: Mild Cognitive Impairment
OR: Odds Ratio
PR: Prevalence Ratio
SCD: Subjective Cognitive Decline
SD: Standard Deviation
SIDAM: Structured Interview for the Diagnosis of Dementia of the Alzheimer Type, Multiinfarct-Dementia and Dementia of other Etiology
VIF: Variance Inflation Factor
