# Supplementary material for: Prevalence and associated factors of subjective cognitive decline (SCD Plus): a cross-sectional analysis of three population-based European cohorts
Source: Alzheimers Res Ther. 2026 May 22;18:130. doi: 10.1186/s13195-026-02087-4 (PMC13220450; doi:10.1186/s13195-026-02087-4)
Supplement: Supplementary file 1 — Supplementary Material 1. [file 13195_2026_2087_MOESM1_ESM.docx]

**Supplementary Material: Zülke et al., Prevalence and associated factors of subjective cognitive decline (SCD Plus): a cross-sectional analysis of three population-based European cohorts**

**Supplementary Table 1: Assessment of SCD Plus criteria across cohorts**

| **Study** | **LIFE** | **ELSA** | **CFAS** |
| --- | --- | --- | --- |
| **SCD+ features** |  |  |  |
| Subjective decline in memory | “Do you feel as if your memory is becoming worse?”  *Yes [1] / No [0]* | “Compared to two years ago, would you say your memory is ...”  *Better now [0]/ about the same [0] / worse [1] than it was then* | “Have you ever had any difficulty with your memory?”  *Yes [1] / No [0]* |
| Worry about memory problems | “If yes, does this worry you?” *No [0] / Yes, this does worry me [1] / Yes, this does worry me very much [1]* | “How would you rate your memory at the present time? Would you say it is…”  *Excellent [0]/ Very good [0]/ Good [0]/ Fair [1] / Poor [1]?* | “Was/is that a problem for you?”  No *[0]*/ Yes, moderate *[1]* / Yes, severe *[1]* |
| Onset of memory problems ≤ 5 years | “Since when have you felt that your memory has deteriorated?”  *Within the last 6 months [1] / more than 6 months ago;*  “How old were you when your memory problems began?”  *age, in years; [1] if onset ≤ 5 years ago* | Onset-criterion accepted as fulfilled for all since the item on subjective memory decline refers to memory two years ago. | “When did you first notice this beginning?”  *< 1 year ago [1] / In the last 1-2 years [1] / In the last 3-4 years [1] / In the last 5-10 years [0] / > 10 years ago [0]* |
| Memory problems confirmed by informant | “Have other people noticed that your memory has gotten worse?”  *No [0]/ [1] Yes, I hear that sometimes / [1] Yes, I hear that often* | (answered by confidant) “Did [he/she] ever have a memory problem?”  *Yes [1] / No[0]* |  |

Questions and answering options assessing SCD Plus criteria across studies. Answering options coded as 0 if not fulfilled, 1 if fulfilled. CFAS I: Cognitive Function and Ageing Study; ELSA: English Longitudinal Study of Ageing; LIFE: LIFE-Adult-Study; SCD: subjective cognitive decline.

**Supplementary Table 2: Harmonization strategy for exclusion criteria and covariates of SCD Plus-criteria**

| **Study** | **LIFE** | **ELSA** | **CFAS I** |
| --- | --- | --- | --- |
| **Construct / variable** |  |  |  |
| Dementia (exclusion criterion) | Major NCD assessed based on DSM-5 criteria; concern of the individual, a knowledgeable informant, or clinician about a significant decline in cognitive function (answering “yes” either to the two questions “Do you feel as if your memory is becoming worse?”, and “If yes, does this worry you?”, or “Have other people noticed that your memory has gotten worse?”;  substantial impairment in cognitive performance: cognitive performance ≥2 SD below age-, sex- and education-specific norms in at least one cognitive test (Trail Making Test A and B, Consortium to Establish a Registry for Alzheimer's Disease (CERAD) Word List Memory, Verbal Fluency Test, CERAD Constructional Praxis Test, Reading the Mind in the Eyes-Test (revised version));  cognitive deficits interfere with independence in everyday activities (ADL; ≥ 2 impaired everyday activities on the SIDAM-ADL scale (Structured Interview for the Diagnosis of Dementia of the Alzheimer Type, Multiinfarct-Dementia and Dementia of other Etiology));  cognitive deficits do not exclusively occur in context of a delirium (SIDAM delirium item); cognitive deficits are not better explained by other mental disorders (e.g., major depression, schizophrenia; (1)). No cases of major NCD at baseline. | Self-reported diagnosis of all-cause dementia or Alzheimer’s disease (yes/no); Informant Questionnaire on Cognitive Decline in the Elderly (IQCODE) | Automated Geriatric Examination for Computer Assisted Taxonomy (AGECAT) algorithm, based on the Geriatric Mental State Examination (2); AGECAT provides organicity score (range: O0 – O5), with values ≥ O3 indicating dementia. |
| Instrumental Activities of Daily Living (severe impairments = exclusion criterion) | Lawton IADL-scale ((3), range: 0-8 points)  Harmonized variable: ≤ 3 points indicating severe impairments in IADL | Difficulties due to a health or memory problem in any of the following activities: (a) preparing a hot meal, (b) shopping for groceries, (c) making telephone calls, (d) taking medications, (e) managing money, such as paying bills and keeping track of expenses (sum score, range: 0-5)  Harmonized variable: ≥ 4 points indicate severe impairments in IADL | Modified Townshend Disability Scale (range: 0-18 points)  Harmonized variable: ≥ 12 points indicate severe impairments in IADL |
| Education | Formal and vocational education, based on the DEGS1-study (4), values ranging from 1.0-7.0;  Harmonized variable: ≤ 2.8 = low; >2.8 < 4.55 = intermediate; ≥ 4.55 = high | Preprocessed information provided by ELSA; categories: "limited high school", "high school graduate", "some college", "college and above”;  Harmonized variable: limited high school = low; high school graduate & some college = intermediate; college and above = high | Years in full-time education;  Harmonized variable: ≤ 9 = low; 10-12 = intermediate; ≥ 13 = high |
| Married/partnership | Self-report of marital status and information on partnership | Self-report of marital status and information on partnership | Self-report of marital status and information on partnership |
| Living alone | Based on information about household size | Based on information about household size | Based on information about household size |
| Depression | CES-D (20-item version; (5)) score ≥ 23 or self-reported diagnosis of depression | CES-D (8-item version; (6)) score ≥ 3 | GMS-AGECAT algorithm (2), clinical case depression, or answering “yes, sounds like depression” to the question “Have you ever consulted a doctor about emotional problems, or problems with your nerves? Perhaps if you were depressed or anxious, or found that you couldn’t enjoy yourself?” |
| Anxiety | GAD-7 (7) score ≥ 10 | Self-reported diagnosis of anxiety | GMS-AGECAT algorithm (2), clinical case anxiety, or answering “yes, sounds like anxiety” to the question “Have you ever consulted a doctor about emotional problems, or problems with your nerves? Perhaps if you were depressed or anxious, or found that you couldn’t enjoy yourself?” |
| Comorbidities | Self-reported history of diabetes mellitus, heart disease, stroke, hypertension, Parkinson’s disease, high cholesterol (yes/no) | Self-reported history of diabetes mellitus, heart disease, stroke, hypertension, Parkinson’s disease, high cholesterol (yes/no) | Self-reported history of diabetes mellitus, heart disease, stroke, hypertension, Parkinson’s disease (yes/no) |
| Smoking | Self-report of current smoking status (yes/no) | Self-report of current smoking status (yes/no) | Self-report of current smoking status (yes/no) |
| Cognitive performance | Composite z-score, based on performance in the Verbal Fluency Test and Trail Making Test A and B | Composite z-score, based on memory (immediate and delayed recall) and executive function (8–10)) | Composite z-score, based on performance in EMSE (11) |
| ***Factors with limited availability across cohorts (not harmonized; used in cohort-specific analyses only)*** | | | |
| Physical activity | IPAQ (12);  IPAQ-categories: low, intermediate, high | Frequency of light, moderate, and vigorous physical activity (PA);  low: no moderate/vigorous PA on a weekly basis; intermediate: moderate PA once a week or light PA more than once a week; high: vigorous PA ≥ once a week, following the approach of (13) | n.a. |
| Alcohol consumption | Alcohol consumption in g/day, based on self-report of frequency of alcohol consumption and average number of alcoholic drinks consumed per occasion | Alcohol consumption in g/day, based on self-report of frequency of alcohol consumption and average number of alcoholic drinks consumed per occasion | Not used due to the high proportion of missing data |
| LDL cholesterol | Determination of LDL cholesterol from serum (mmol/l) | Determination of LDL cholesterol from blood (mmol/l) | n.a. |
| Sleep problems | Pittsburgh Sleep Quality Index (PSQI; (14)); range: 0-21 points, higher scores indicate worse sleep quality |  | “Have you had any trouble sleeping recently?” (yes / no); “yes” is rated as a sleeping problem |
| Hearing problems | n.a. | “Is your hearing… (excellent / very good / good / fair / poor)?”; rating of hearing as “fair” or “poor” is rated as a hearing problems | “Do you suffer from hearing problems which interfere with day-to-day living?” (yes / no); “yes” is rated as hearing problems |
| ApoE genotype | ApoE genotyping was performed by a melting curve analysis on a LightCycler® 480 (Hoffman-LaRoche, Basel, Switzerland) according to the method of (15).  ≥ 1 ApoE ε4 allele = ApoE ε4 carrier | n.a. | ApoE genotyping performed by PCR amplification of the ApoE gene region followed by HhaI restriction enzyme digestion (PCR-RFLP), allowing differentiation of ε2, ε3, and ε4 alleles based on fragment sizes (16);  ≥ 1 ApoE ε4 allele = ApoE ε4 carrier |
| Thyroid disease | Self-reported diagnosis of thyroid disease (any) | n.a. | Self-reported diagnosis of thyroid disease (underactive current/past, overactive current/past, other/non-specific current/past) |
| Exposure to air pollution | Air pollution data collected and provided through the city of Leipzig; air pollution assessed using 5-year means (2010-2014) of fine particles with an aerodynamic diameter of < 2.5 μm particulate matter (PM_2.5_) | n.a. | n.a. |
| Personality traits | Personality Adjective List (16 AM), assessing the Big 5 domains neuroticism, openness, agreeableness, conscientiousness, extraversion (17) | n.a. | n.a. |

ADL: activities of daily living; AGECAT: Automated Geriatric Examination for Computer Assisted Taxonomy; APOE: Apolipoprotein E; CES-D: Centre for Epidemiological Studies - Depression Scale; CFAS: Cognitive Function and Ageing Study; DEGS1: German Health Interview and Examination Survey for Adults; DSM-V: Diagnostic and Statistical Manual of Mental Disorders, 5^th^ edition; ELSA: English Longitudinal Study of Ageing; EMSE: Extended Mental State Exam; GAD: Generalized Anxiety Disorder scale; GMS-AGECAT: Geriatric Mental State - Automated Geriatric Examination for Computer Assisted Taxonomy; IADL: Instrumental Activities of Daily Living; LDL: low-density lipoprotein; n.a.: not applicable; NCD: neurocognitive disorder; SD: standard deviation

**Supplementary Table 3: Multivariable associations with SCD Plus in the total sample (Poisson regression), excluding participants with depression and/or anxiety**

| Variable | PR | 95% CI | *p* |
| --- | --- | --- | --- |
| Age | 1.00 | 1.00; 1.01 | .448 |
| Female sex | 1.05 | 0.98; 1.12 | .159 |
| Education intermediate (ref.: low) | 1.22 | 0.94; 1.59 | .132 |
| Education high | 1.34 | 1.02; 1.76 | **.034** |
| Married/partnership (ref.: single/divorced/widowed) | 1.15 | 0.99; 1.33 | .065 |
| Living alone (ref.: multi-person household) | 1.02 | 1.01; 1.04 | **<.001** |
| Hypertension | 1.07 | 1.04; 1.11 | **<.001** |
| Diabetes mellitus | 1.07 | 1.06; 1.09 | **<.001** |
| Heart disease | 1.10 | 1.01; 1.20 | **.026** |
| Parkinson’s disease | 1.35 | 1.26; 1.45 | **<.001** |
| History of stroke | 1.11 | 1.03; 1.20 | **.005** |
| Smoking | 0.84 | 0.80; 0.89 | **<.001** |
| Cognitive performance (composite z-score) | 0.89 | 0.84; 0.94 | **<.001** |

CI: confidence interval; PR: prevalence ratio; ref.: reference

**Supplementary Table 4:** **Multivariable associations with SCD Plus in the total sample, mixed effects logistic regression model with study-level intercept**

| Variable | OR | 95% CI | *p* |
| --- | --- | --- | --- |
| Age | 1.01 | 1.01; 1.02 | **<.001** |
| Female sex | 1.08 | 1.00; 1.16 | **.037** |
| Education intermediate (ref.: low) | 1.16 | 1.07; 1.26 | **<.001** |
| Education high | 1.20 | 1.08; 1.33 | **.001** |
| Married/partnership (ref.: single/divorced/widowed) | 1.11 | 0.98; 1.27 | .107 |
| Living alone (ref.: multi-person household) | 0.94 | 0.82; 1.07 | .333 |
| Depression | 1.57 | 1.40; 1.76 | **<.001** |
| Anxiety | 1.75 | 1.38; 2.22 | **<.001** |
| Hypertension | 1.02 | 0.95; 1.09 | .626 |
| Diabetes mellitus | 1.04 | 0.93; 1.17 | .466 |
| Heart disease | 1.15 | 1.06; 1.25 | **.001** |
| Parkinson’s disease | 1.44 | 0.93; 2.23 | .100 |
| History of stroke | 1.25 | 1.06; 1.46 | **.007** |
| Smoking | 0.83 | 0.75; 0.91 | **<.001** |
| Cognitive performance (composite z-score) | 0.93 | 0.88; 0.98 | **.008** |
| Study (random intercept) | 0.10 | 0.02; 0.53 |  |

CI: confidence interval; OR: odds ratio; ref.: reference

**Supplementary Table 5: Multivariable associations with SCD Plus in the total sample (Poisson regression), fully harmonized SCD Plus criteria**

| Variable | PR | 95% CI | *p* |
| --- | --- | --- | --- |
| Age | 1.00 | 0.98; 1.02 | .903 |
| Female sex | 1.18 | 0.88; 1.59 | .265 |
| Education intermediate (ref.: low) | 1.53 | 0.95; 2.44 | .081 |
| Education high | 1.84 | 1.18; 2.88 | **.007** |
| Married/partnership (ref.: single/divorced/widowed) | 1.07 | 0.82; 1.41 | .616 |
| Living alone (ref.: multi-person household) | 1.09 | 0.87; 1.37 | .450 |
| Depression | 1.31 | 1.09; 1.56 | **.003** |
| Anxiety | 1.64 | 1.37; 1.97 | **<.001** |
| Hypertension | 1.07 | 0.95; 1.21 | .280 |
| Diabetes mellitus | 1.15 | 1.08; 1.23 | **<.001** |
| Heart disease | 1.12 | 1.03; 1.21 | **.005** |
| Parkinson’s disease | 1.43 | 0.96; 2.15 | 0.82 |
| History of stroke | 1.21 | 1.14; 1.29 | **<.001** |
| Smoking | 0.93 | 0.84; 1.03 | .177 |
| Cognitive performance (composite z-score) | 0.84 | 0.70; 1.01 | .061 |

CI: confidence interval; PR: prevalence ratio; ref.: reference

Literature Cited

1. Luck T, Then FS, Schroeter ML, Witte V, Engel C, Loeffler M et al. Prevalence of DSM-5 mild neurocognitive disorder in dementia-free older adults: results of the population-based LIFE-adult-study. The American Journal of Geriatric Psychiatry 2017; 25(4):328–39.

2. Copeland JR, Dewey ME, Griffiths-Jones HM. A computerized psychiatric diagnostic system and case nomenclature for elderly subjects: GMS and AGECAT. Psychological medicine 1986; 16(1):89–99.

3. Lawton M, Brody E, Médecin U. Instrumental activities of daily living (IADL). Gerontologist 1969; 9:179–86.

4. Lampert T, Kroll L, Müters S, Stolzenberg H. Messung des sozioökonomischen Status in der Studie zur Gesundheit Erwachsener in Deutschland (DEGS1). Bundesgesundheitsblatt-Gesundheitsforschung-Gesundheitsschutz 2013; 56(5):631–6.

5. Radloff LS. The CES-D scale: A self-report depression scale for research in the general population. Applied psychological measurement 1977; 1(3):385–401.

6. Karim J, Weisz R, Bibi Z, Ur Rehman S. Validation of the eight-item center for epidemiologic studies depression scale (CES-D) among older adults. Current Psychology 2015; 34(4):681–92.

7. Spitzer RL, Kroenke K, Williams JBW, Löwe B. A brief measure for assessing generalized anxiety disorder: the GAD-7. Archives of internal medicine 2006; 166(10):1092–7.

8. Taylor R, Conway L, Calderwood L, Lessof C, Cheshire H, Cox K et al. Health, wealth and lifestyles of the older population in England: The 2002 English Longitudinal Study of Ageing: Technical Report; 2007 [cited 2025 Dec 17]. Available from: URL: https://ifs.org.uk/sites/default/files/output_url_files/w1_tech.pdf.

9. Yin J, Lassale C, Steptoe A, Cadar D. Exploring the bidirectional associations between loneliness and cognitive functioning over 10 years: the English longitudinal study of ageing. Int J Epidemiol 2019; 48(6):1937–48.

10. Maharani A, Tampubolon G. National economic development status may affect the association between central adiposity and cognition in older adults. PloS one 2016; 11(2):e0148406.

11. Huppert FA, Cabelli ST, Matthews FE. Brief cognitive assessment in a UK population sample -- distributional properties and the relationship between the MMSE and an extended mental state examination. BMC Geriatr 2005; 5:7.

12. Hagströmer M, Oja P, Sjöström M. The International Physical Activity Questionnaire (IPAQ): a study of concurrent and construct validity. Public Health Nutr 2006; 9(6):755–62.

13. Hamer M, Molloy GJ, Oliveira C de, Demakakos P. Leisure time physical activity, risk of depressive symptoms, and inflammatory mediators: the English Longitudinal Study of Ageing. Psychoneuroendocrinology 2009; 34(7):1050–5.

14. Buysse DJ, Reynolds CF, Monk TH, Hoch CC, Yeager AL, Kupfer DJ. Quantification of subjective sleep quality in healthy elderly men and women using the Pittsburgh Sleep Quality Index (PSQI). Sleep 1991; 14(4):331–8.

15. Aslanidis C, Schmitz G. High-speed apolipoprotein E genotyping and apolipoprotein B3500 mutation detection using real-time fluorescence PCR and melting curves. Clinical Chemistry 1999; 45(7):1094–7.

16. Hixson JE, Vernier D. Restriction isotyping of human apolipoprotein E by gene amplification and cleavage with HhaI. Journal of lipid research 1990; 31(3):545–8.

17. Herzberg PY, Brähler E. Assessing the Big-Five Personality Domains via Short Forms. European Journal of Psychological Assessment 2006; 22(3):139–48.
